# Omega-3 fatty acid EPA improves regenerative capacity of mouse skeletal muscle cells exposed to saturated fat and inflammation

**DOI:** 10.1007/s10522-016-9667-3

**Published:** 2016-11-18

**Authors:** Amarjit Saini, Adam P. Sharples, Nasser Al-Shanti, Claire E. Stewart

**Affiliations:** 1 Department of Laboratory Medicine, Clinical Physiology, Karolinska Institutet, and Unit of Clinical Physiology, Karolinska University Hospital, 141 86 Stockholm, Sweden; 2 Stem Cells, Ageing and Molecular Physiology (SCAMP) Unit, Exercise Metabolism and Adaptation Research Group, Research Institute for Sport & Exercise Sciences, School of Sport and Exercise Sciences, Liverpool John Moores University, Life Science Building, Byrom Street Campus, Liverpool, L3 3AF UK; 3 Neuromuscular and Skeletal Ageing Research Group, Healthcare Science Research Institute, Manchester Metropolitan University, Oxford Road, Manchester, M1 5GD UK

**Keywords:** Palmitate, TNF, Cytokines, Sarcopenia, Sarcopenic obesity

## Abstract

Sarcopenic obesity is characterised by high fat mass, low muscle mass and an elevated inflammatory environmental milieu. We therefore investigated the effects of elevated inflammatory cytokine TNF-α (aging/obesity) and saturated fatty acid, palmitate (obesity) on skeletal muscle cells in the presence/absence of EPA, a-3 polyunsaturated fatty acid with proposed anti-inflammatory, anti-obesity activities. In the present study we show that palmitate was lipotoxic, inducing high levels of cell death and blocking myotube formation. Cell death under these conditions was associated with increased caspase activity, suppression of differentiation, reductions in both creatine kinase activity and gene expression of myogenic factors; IGF-II, IGFBP-5, MyoD and myogenin. However, inhibition of caspase activity via administration of Z-VDVAD-FMK (caspase-2), Z-DEVD-FMK (caspase-3) and ZIETD-KMK (caspase 8) was without effect on cell death. By contrast, lipotoxicity associated with elevated palmitate was reduced with the MEK inhibitor PD98059, indicating palmitate induced cell death was MAPK mediated. These lipotoxic conditions were further exacerbated in the presence of inflammation via TNF-α co-administration. Addition of EPA under cytotoxic stress (TNF-α) was shown to partially rescue differentiation with enhanced myotube formation being associated with increased MyoD, myogenin, IGF-II and IGFBP-5 expression. EPA had little impact on the cell death phenotype observed in lipotoxic conditions but did show benefit in restoring differentiation under lipotoxic plus cytotoxic conditions. Under these conditions Id3 (inhibitor of differentiation) gene expression was inversely linked with survival rates, potentially indicating a novel role of EPA and Id3 in the regulation of apoptosis in lipotoxic/cytotoxic conditions. Additionally, signalling studies indicated the combination of lipo- and cyto-toxic effects on the muscle cells acted through ceramide, JNK and MAPK pathways and blocking these pathways using PD98059 (MEK inhibitor) and Fumonisin B1 (ceramide inhibitor) significantly reduced levels of cell death. These findings highlight novel pathways associated with in vitro models of lipotoxicity (palmitate-mediated) and cytotoxicity (inflammatory cytokine mediated) in the potential targeting of molecular modulators of sarcopenic obesity.

## Introduction

The two greatest epidemiological trends of modern times are the rapidly advancing age of the worldwide population and the obesity epidemic. Ageing is accompanied by changes in body composition characterised by a relative decline in muscle mass (Aniansson et al. 1983) and an increase in fat mass (Lara-Castro et al. 2002), a condition termed sarcopenic obesity (SO) (Roubenoff 2004; Zamboni et al. 2008). The prevalence of sarcopenic obesity is thought to account for 25% of the population aged over 60 (Batsis et al. 2016) with a greater prevalence reported in men (42.9%) than women (18.1%) (Batsis et al. 2014). A recent meta-analysis including 35,287 participants and 14,306 deaths, suggested that individuals with SO have a 24% increased risk of all-cause mortality compared to those without SO (Tian and Xu 2016). With a global rise in ageing and obesity levels, it is likely that this condition will represent a growing clinical and financial burden for the health care systems of developed countries (Sousa et al. 2016).

Independently, excess adiposity and reduced muscle mass lead to adverse metabolic effects (e.g., hypertension, dyslipidaemia, insulin resistance). A combination of increased adiposity and reduced lean mass, engendered by ageing, may therefore contribute further to the worsening of metabolic impairments. Furthermore, the combination of low muscle mass and strength with obesity are further associated with a pro-inflammatory state, where adipocytes actively secrete increased concentrations of pro-inflammatory cytokines (Fantuzzi 2005; Mohamed-Ali et al. 1998), which stimulate further muscle catabolism (Hotamisligil 1999a, b) thereby activating a vicious cycle culminating in accelerated sarcopenia, additional adiposity and ultimately, physical disability. With age there is also a local increase in the production of inflammatory cytokines by skeletal muscle (Greiwe et al. 2001; Leger et al. 2008). Elevated levels of cytokines have been shown to predict accelerated decline of muscle strength and increased risk of early mortality in older individuals (Cappola et al. 2003; Ferrucci et al. 1999; Harris et al. 1999). However, the underlying mechanisms of morbidity and early mortality are poorly understood.

Circulating free fatty acids (FFAs) are elevated in obese adults (Reaven et al. 2004) and in aged muscle with increased fat infiltration, suggesting a connection between a gain in fat mass, muscle triglyceride content and inflammation with age (Zamboni et al. 2008). In particular, the saturated free fatty acid, Palmitate (C_16_H_32_O_2_) reduces insulin-stimulated phosphorylation of Akt (pAkt)—a key molecule in the insulin-like growth factor (IGF) signalling pathway, which acts potently to regulate protein synthesis, skeletal muscle regeneration and differentiation (Egerman and Glass 2014). In contrast, studies have described the potential anti-inflammatory health benefits of n-3 polyunsaturated fatty acids such as eicosapentaenoic acid (C_20_H_30_O_2_; EPA) with potential benefits for atrophying skeletal muscle (Babcock et al. 2002; Magee et al. 2008). Indeed, in murine models of cachexia, EPA treatment caused a reduction in the rate of skeletal muscle protein loss (Tisdale and Dhesi 1990; Whitehouse et al. 2001; Whitehouse and Tisdale 2001) which was associated with a downregulation of the ubiquitin–proteasome pathway (Whitehouse and Tisdale 2001). Additionally, EPA treatment has been shown to attenuate the proteolytic and apoptotic effects of cachectic factors in fully differentiated mouse C2C12 myotubes (Smith et al. 1999; Smith and Tisdale 2003).

Adult skeletal muscle is terminally differentiated, therefore any repair and regeneration of the muscle is dependent on resident satellite or infiltrating stem cells (Cooper et al. 1999). The ability of these cells to replicate and to fuse is dependent on the local growth factor/cytokine environment (Saini et al. 2006, 2009; Sharples et al. 2015; Sharples and Stewart 2011; Sharples et al. 2016). Furthermore, altered cytokine levels have relevance in the pathogenesis of atrophy by impeding regenerative processes and stimulating protein catabolism. It is therefore conceivable, in conditions of SO, that alterations in local levels of FFAs and concomitant changes in growth factors and cytokines may contribute to declining muscle mass and strength. Previous findings led us to hypothesise that palmitate potentiates whereas EPA protects against skeletal muscle damage induced by pro-inflammatory TNF-α (Sabin et al. 2007) and that EPA would further reduce the lipotoxic and cytotoxic effect of elevated palmitate plus TNF-α. The present study was designed to investigate the cellular and molecular mechanisms of lipotoxic (palmitate) and cytotoxic (TNF-α) combinations on skeletal muscle cell survival and differentiation in the absence or presence of EPA. The ultimate aim was therefore to improve muscle cell survival and differentiation using EPA as a myoprotective agent, and elucidate the underlying mechanisms of action.

## Materials & methods

### Materials

All cell and tissue culture media and supplements were purchased as sterile or were filter sterilised through a 0.20 µM filter. Heat-inactivated (hi) foetal bovine serum (FBS) and hi new born calf serum (NCS) were purchased from Gibco (Paisley, Scotland); hi horse serum (HS) from TCS Biosciences (Corby, England); penstrep (penicillin and streptomycin) and trypsin from Bio Whittaker (Wokingham, England); l-glutamine from BDH (Poole, England), gelatine from Sigma (St. Louis, USA). Glassware, distilled water (dH_2_O) and phosphate buffered saline (PBS, Oxoid (Hampshire, England) were autoclaved prior to use. Sterile media were stored at 4 °C and used within 2 months. Plasticware were purchased as sterile from Greiner Bio-one, (Kremsmunster, Austria) unless otherwise stated. EPA purchased from IDS Ltd (Boldon, Tyne & Wear, UK) and Palmitate (Sigma-Aldrich, Poole, UK) were complexed with fatty-acid free bovine serum albumin (BSA) (Sigma-Aldrich, Poole, UK). Recombinant human TNF-α was purchased from Calbiochem (Nottingham, England); TRIZOL reagent (Invitrogen, Life Technologies), propidium iodide (PI) stain from BD Biosciences (San Diego, USA) and creatine kinase assay kits from Catachem (Bridgeport, USA). Recombinant human IGF-I was purchased from GroPep (Adelaide, Australia). BCA™ protein quantitation reagents were obtained from Pierce Chemicals (Chester, England, UK).

### Cell culture

C2 mouse skeletal myoblasts (passages 8–12) (Yaffe and Saxel 1977) were grown in T75 flasks in a humidified 5% CO_2_ atmosphere at 37 °C in growth medium (GM), composed of: DMEM with Glutamax supplemented with 10% hiFBS, 10% hiNCS, penstrep and additional l-glutamine, at final concentrations of 10000 U ml^−1^ and 2 mM respectively, until 80% confluency was attained. Experiments and differentiation were initiated following washing with phosphate buffered saline (PBS), by transferring to low serum-containing differentiation medium (DM; DMEM plus glutamax, supplemented with 2% HS, penstrep and additional l-glutamine (supplemented as above)) in the absence or presence of specific cytokines, growth factors, lipids and signal inhibitors described below. C2 myoblasts, upon serum withdrawal, are able to undergo spontaneous differentiation into myotubes and do not require growth factor addition to stimulate the process (Florini et al. 1991). Adherent cells following trypsinisation, and detached cells in the supernatant, were counted using a haemocytometer in the presence of trypan blue dye (Bio Whittaker, Wokingham, England) to assess viability and cell number.

### Cell treatments

Six-well plates were pre-coated with 0.2% gelatin (Sigma) for 5 min at room temperature. Following removal of excess gelatin, cells were seeded at 1 × 10^5^ cells ml^−1^ in GM. On attaining 80% confluency, cells were washed twice with PBS and transferred to DM for 30 min at 37 °C. The end of this 30 min equilibration time-point was denoted time 0.

### Interactions of FFAs with TNF-α and IGF-I

To evaluate the effects of FFAs on myoblast death and myogenic differentiation, DM supplemented with EPA (50 µM) (Magee et al. 2008) or Palmitate (750 µM) (Sabin et al. 2007) was added to cell cultures at time-point 0 for 1-h pre-incubation prior to vehicle, TNF-α and/or IGF-I addition (doses described below). EPA was first complexed with fatty-acid free BSA. Briefly, EPA stock solutions (50 mM) were prepared in absolute ethanol and stored at −20 °C in a glass vial in the dark. Working solutions were prepared by adding the required volume of EPA stock solution to pre-warmed (37 °C) DMEM containing 4% (w/v) fatty acid-free BSA. Dilutions were maintained at 37 °C for at least 1-h before their addition to cell cultures. The final concentration of ethanol in cultures was below 0.1%. Medium containing palmitate was generated based on a previously published method (Schmitz-Peiffer et al. 1999). Briefly, FFA-depleted albumin was dissolved in serum free media (SFM) to make a 20% albumin solution. 75 mM palmitate in ethanol was mixed with the albumin solution and made up with 2% media to generate a final FFA-containing media with 5% albumin. Serial dilutions were performed, prior to adding 2% HS containing media in order to maintain the final albumin concentration at 5%. Final solutions were filter-sterilised before use. Doses of palmitate were based on previously published studies on our data in human skeletal muscle cells and of others in C2C12 cells (Sabin et al. 2007; Schmitz-Peiffer et al. 1999). Control wells were dosed with media containing 5% albumin and no FFAs. The pH of the media did not differ significantly with the addition of the complexed fatty acid. EPA and palmitate were delivered 30-min following change to DM. Where EPA and palmitate were co-incubated, both were simultaneously administered. On cytokine and growth factor interactions at time-point 1-h after EPA and/or Palmitate treatment, myoblasts received TNF-α (1.25 or 10 ng ml^−1^), IGF-I (1.5 ng ml^−1^) or TNF-α (10 ng ml^−1^) + IGF-I (1.5 ng ml^−1^). Following 36 and 72 h incubations, RNA was extracted for real time RT-PCR. For cell counting and flow cytometric studies, cells were trypsinised 48 h following dosing. For studies involving creatine kinase assays, cells were harvested following 72 h incubation, as described below.

### TNF-α and IGF-I inhibitor studies

Where inhibitor studies were performed, upon reaching 80% confluency, cells were washed twice in PBS and were immediately transferred into DM. After 30-min, wells were treated in the absence or presence of the MEK inhibitor, PD98059 (20 µM) (Al-Shanti et al. 2008; Saini et al. 2008, 2010, 2012, ) or in the absence of presence of fungal toxin fumonisin B1 (FB1) (an inhibitor of ceramide synthase (20 µM)) as previously demonstrated (Mebarek et al. 2007). After 1-h myoblasts were administered with EPA and/or Palmitate for 1-h at 37 °C. Cultures were subsequently spiked with TNF-α (1.25 or 10 ng ml^−1^) or IGF-I (1.5 ng ml^−1^) or TNF-α (10 ng ml^−1^) + IGF-I (1.5 ng ml^−1^).

### Primer design and synthesis

Optimal primer designs are essential to ensure that only a single PCR product is amplified using quantitative Real Time PCR based SYBR Green technology, Web-based software from Invitrogen was used to design primers (Table 1) which were further analysed by Sigma- Genosys software. The primers were designed to yield products spanning exon–exon boundaries to prevent possible genomic DNA-contamination. Sequence homology searches against the database of GenBank showed that these primers matched only the sequence against which they were designed. Primers were synthesised by Sigma-Genosys (Suffolk, UK) which are compatible with real-time PCR without further purification.

**Table 1:** Primer sequences

Target gene	Primer sequence (5′–3′)	Ref. seq number	Primer length (product length) bp
IGF-II	F: TCTCATCTCTTTGGCCTTCG R: AAGCAGCACTCTTCCACGAT	NM_010514	20 (product length 180) 20
Id3	F: AGCGTGTCATAGACTACATCCTC R: TCCTCTTGTCCTTGGAGATCAC	NM_008321	23 (product length 136) 22
IGFBP-5	F: GACGACCCAGTCCAAGTTTGTGG R: GAATCCTTTGCGGTCACAGT	NM_010518	20 (product length 193) 20
RP-IIb (polr2b)	F: GGTCAGAAGGGAACTTGTGGTAT R: GCATCATTAAATGGAGTAGCGTC	NM_153798.1	23(product length 197) 23
Myogenin QuantiTect primer assay	QiagenQT00112378	NM_031189	
MyoD QuantiTect primer assay	QiagenQT00101983	NM_010866	

### RNA extraction and qRT-PCR

Following cell treatments and incubations, differentiation medium was aspirated, cells were washed with PBS and lysed with 200 µl TRIZOL reagent. RNA was extracted from TRIZOL homogenates according to the manufacturer’s instructions (Invitrogen, CA, USA). Thirty ng RNA was used per reaction. Following RNA isolation, qRT-PCR was performed with the Chromo4 Detection System (Biorad Laboratories, Hercules, CA, USA), supported by Opticon Monitor Version 3.1 analysis software and using Biorad iScript™ One-Step RT-PCR Kit with SYBR Green, (Hercules, CA, USA) according to the manufacturers’ instructions. The amplification program included an initial denaturation step at 95 °C for 10 min, followed by 40 cycles of denaturation at 95 °C for 15 s, and annealing and extension at 60 °C for 1 min. SYBR Green fluorescence was measured after each extension step, and the specificity of amplification was evaluated by melting curve analyses. The relative gene expression levels were calculated using the comparative Ct (^ΔΔ^C_t_) method (Schmittgen and Livak 2008), where the relative expression is calculated as 2^−ΔΔ^Ct and where C_t_ represents the threshold cycle. The calibrator condition for 2^−ΔΔ^Ct calculation was DM control at 36 h. The reference gene was RP-IIb/Polr2b) Table 1, and was stable (Ct value mean ± SD = 24.482 ± 0.59) across experimental conditions. Every sample was run in three separate experiments in duplicate.

### Analysis of cell cycle by flow cytometry

Following 48 h incubation, adherent cells were trypsinised in 0.2 ml of 0.5% trypsin/0.02% EDTA solution and were pooled with detached cells, resuspended and washed in PBS prior to fixing at −20 °C in 70% ethanol. After a minimum of 24-h, cells were pelleted (200×*g* for 5-min), washed in PBS (three times 200×*g* for 5-min) and resuspended with gentle vortexing in propidium iodide labelling buffer (50 µg ml^−1^ propidium iodide, 0.1% sodium citrate, 20 µg ml^−1^ ribonuclease A, 0.3% Nonidet P-40, pH 8.3) at approximately ~1 × 10^6^ cells ml^−1^. Cells were stored in the dark at 4 °C for 30-min, prior to assaying at room temperature, using a Becton–Dickinson FACSCalibur flow cytometer. Data were analysed using Cell Quest software (Becton–Dickinson, Oxford, England).

### Analysis of intracellular caspase detection by flow cytometry

Following 48 h incubation, myoblasts were stained directly by adding 10 ml of ApoStat (R & D Systems, Abingdon, UK) per 1 ml culture volume at 37 °C. After the staining period, cells were harvested into 5 ml tubes, centrifuged at 500×*g* for 5 min and washed once with 4 ml PBS to remove unbound reagent. Cells were resuspended in 500 µl of PBS for flow cytometric analyses. Induced and non-induced cells were observed on a side scatter versus forward scatter linear dot plot to identify and gate cells of interest. Fluorescein detection was collected on the FL1 channel (employing an argon laser at 488 nm).

### Flow cytometry: cytometric bead array (CBA) for quantification of phosphorylated proteins

BD™ CBA is a flow cytometry application based on phycoerythrin (PE) antibody-coated beads for simultaneous quantification of multiple proteins, including intracellular phosphorylated signalling proteins (Schubert et al. 2009) in single samples. Cells were washed at 4 °C in PBS and lysed using 1 × lysis buffer provided in the Cell Signalling Master Buffer Kit (BDTM CBA). The cell lysates were denatured at 100 °C and dispersed using a 26- gauge needle. A protein assay (BCA™) was performed to determine protein concentrations of individual samples. Cell lysates were stored at −70 °C until required for the CBA. Samples were thawed and added to the assay diluent provided in the Cell Signalling Master Buffer Kit (15 µg/sample). Standards were prepared using a stock of recombinant protein (50,000 U ml^−1^) contained in the BD™ CBA Cell Signalling Flex Set (JNK). Serial dilutions of the top standard (1000 U ml^−1^) were performed. All samples were incubated in the dark for 2 h prior to further investigation. PE detection reagent was added to each sample and incubated at RT (protected from light) for a further 1 h. The samples were washed in wash buffer (provided in CBA kits) and centrifuged at 300×*g* for 5 min. Excess liquid was removed and 300 µl fresh wash buffer was added to each pelleted sample, prior to gentle vortexing and analyses using Cell Quest Pro (Becton–Dickinson) on a BD™ FACS Calibur as per manufacturer instructions. Data were uploaded from Cell Quest Pro, filtered using FCS FilterTM and analysed using FCAP array software (Hungary Software Ltd., for BD Biosciences).

### Creatine kinase assay

Cells were treated as described above, washed twice with PBS and lysed in 0.2 ml of 50 mM Tris-MES, pH 7.8, 1% Triton X-100 (TMT buffer). Samples were stored at −80 °C, and assayed within 2 weeks of harvesting using a commercially available kit (Catachem CK assay) according to manufacturer’s instructions. Enzymatic activity was normalised to total protein content as determined by the BCA™ assay (Pierce, Rockford, IL, USA).

### Statistical analysis

Data were analysed using Microsoft Excel version 7.0 and SPSS version 11.5 software and GraphPad version 5.0 software. Results are presented as the mean ± standard error of the mean (SEM). Statistical significance was determined using one-way analysis of variance with multiple post hoc analyses. Results were considered statistically significant when P < 0.05. All experiments were performed at least 3 times in duplicate, unless otherwise stated.

## Results

### Palmitate increases cell death and reduces differentiation, which is improved with EPA administration

**Fig. 1:**
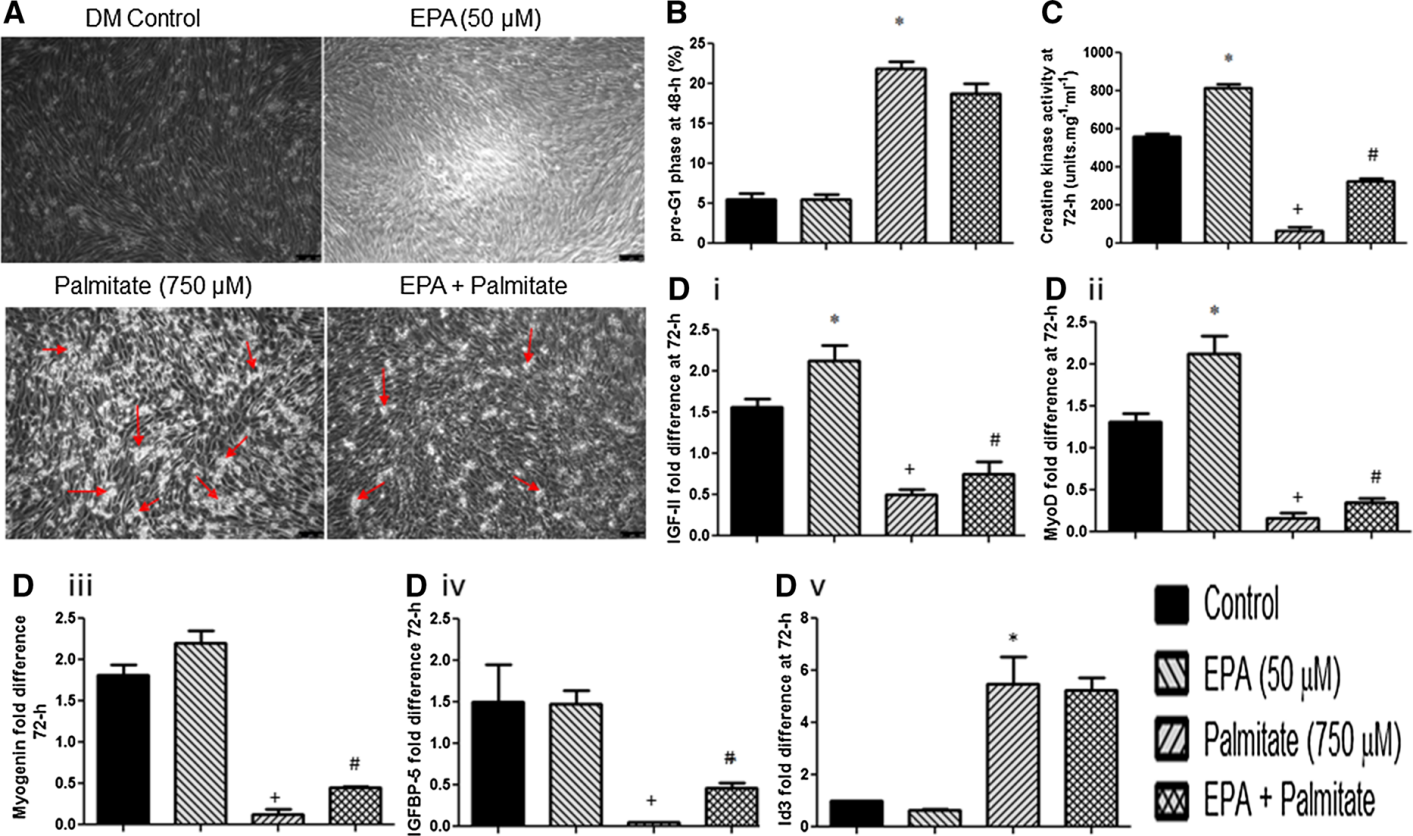
Palmitate increases death and reduces differentiation in myoblasts, with differentiation improved with EPA administration. **a** At a microscopic level, an increase in cell death was observed with palmitate that appeared reduced by a co-incubation with EPA. *Arrows* indicate congregations of dead cells. **b** Flow cytometric data confirmed, cell death was significantly increased (vs. DM and EPA) by a single dose of palmitate (22 ± 0.9%; indicated by *). With the addition of EPA there was a reduction in cell death versus palmitate alone (19 ± 0.9 vs. 22 ± 0.9%) however, significance was not achieved. **c** EPA alone significantly increased biochemical differentiation (creatine kinase (CK) activity) (817 ± 17 vs. 562 ± 16 units mg^−1^ ml^−1^ in DM control, indicated by *). With palmitate, CK activity was significantly reduced versus control DM and EPA alone (68 ± 19 units mg ml^−1^; P < 0.05 vs. DM and vs. EPA; indicated by +). Co-incubating palmitate and EPA, resulted in significantly elevated creatine kinase activity compared to palmitate alone, increasing to 325 ± 16 units mg ml^−1^ (indicated by #). **d** Gene expression of IGF-II (**di**), MyoD (**dii**), myogenin (**diii**) and IGFBP5 (**div**) was up-regulated at 72 h (relative to DM 36 h) in response to differentiation in control conditions (DM treatment). EPA resulted in higher gene expression compared with DM for IGF-II (2.13 ± 0.18 vs. 1.56 ± 0.1; **di** indicated by *) and MyoD (2.13 ± 0.21 vs. 1.31 ± 0.11; P < 0.05; **dii** indicated by *). In line with its ability to suppress differentiation, palmitate significantly reduced all positive gene transcript regulators of differentiation at 72 h; IGF-II (0.51 ± 0.07; P < 0.05), MyoD (0.16 ± 0.06; P < 0.05), myogenin (0.13 ± 0.07; P < 0.05) and IGFBP-5 (0.05 ± 0.005; P < 0.05) **di**–**iv** respectively; indicated by +, and significantly increased inhibitor of differentiation 3 (Id3; **dv**) expression levels (5.51 ± 1.04) compared with DM (1.01 ± 0.001; P < 0.05; indicated by *). Addition of EPA to palmitate versus palmitate alone elevated IGF-II 1.5 fold (0.75 ± 0.15 vs. 0.51 ± 0.07; P > 0.05; **di** indicated by #), MyoD 2.2 fold (0.35 ± 0.05 vs. 0.16 ± 0.06; P > 0.05; **dii** indicated by #), Myogenin 3.5 fold (0.45 ± 0.02 vs. 0.13 ± 0.07; P < 0.05; **diii** indicated by #) and IGFBP-5 9.2 fold (0.46 ± 0.07 vs. 0.05 ± 0.005; P < 0.05; **div** indicated by #). Data are presented as Mean ± SEM (n = 3, in duplicate for each group)

Initial experiments assessed the effects of EPA (50 µM) and palmitate (750 µM) on C2 skeletal muscle cells. Basal myoblast death was the same in control DM-treated and EPA-treated cells, whereas increased levels of death were evident following palmitate administration (Fig. 1a). At a microscopic level, this increase in cell death with palmitate appeared to be reduced by a co-incubation with EPA (Fig. 1a, bottom panel). Wishing to substantiate these morphological findings, apoptotic cell death was assessed using FLOW cytometry (Fig. 1b). Data confirmed basal cell death (pre-G1 phase cells) in the presence of DM (5.6 ± 0.7%), which was unaltered by pre-treatment with EPA (5.5 ± 0.6%; p = N.S.; Fig. 1b). By contrast, cell death was significantly increased (vs. DM and EPA) by a single dose of palmitate (22 ± 0.9%; P < 0.05; Fig. 1b, indicated by *). However, in contrast to the morphological data, analysis of pre G1 fragmentation by flow cytometry illustrated that when palmitate was co-incubated with EPA, there was only a small non-significant reduction in cell death versus palmitate alone (19 ± 0.9 vs. 22 ± 0.9%; Fig. 1b).

Having monitored the effect of EPA and palmitate on cell death, we next investigated their impact on muscle cell differentiation. We hypothesised EPA would have little effect and palmitate would reduce differentiation versus DM control. Creatine kinase activity, a biochemical marker of myoblast differentiation, was elevated after 72 h in control samples (562 ± 16 units mg ml^−1^; Fig. 1c). Myotube formation was also observed in myoblasts receiving EPA, which displayed maximal creatine kinase activity (817 ± 17 units mg ml^−1^) and was significantly higher (P < 0.05) versus DM alone (Fig. 1c). In myoblasts treated with palmitate, creatine kinase activity was reduced versus control DM and EPA alone (68 ± 19 units mg ml^−1^; P < 0.05 vs. DM and vs. EPA, Fig. 1c, indicated by +). Co-incubating palmitate and EPA resulted in significantly elevated creatine kinase activity compared to palmitate alone (P < 0.05) increasing to 325 ± 16 units mg ml^−1^, Fig. 1c, indicated by #), suggesting EPA not only enabled differentiation basally, but was capable of partially rescuing the inhibitory impact of palmitate on differentiation.

To further understand the regulation of myotube formation, the gene expression profiles of key myogenic markers were analysed (Fig. 1d). Importantly, IGF-II, IGFBP-5, MyoD and myogenin expression (Fig. 1d) were all up-regulated at 72 h in response to DM treatment. Since EPA increased biochemical differentiation, it was subsequently hypothesised that expression of these modulators of differentiation would also be increased under these culture conditions. Indeed, EPA resulted in higher gene expression relative to DM for IGF-II (2.13 ± 0.18 vs. 1.56 ± 0.1 e; P < 0.05, Fig. 1di indicated by *) and MyoD (2.13 ± 0.21 vs. 1.31 ± 0.11; P < 0.05; Fig. 1dii, indicated by *). By contrast, there was no difference in Myogenin (2.2 ± 0.16 vs. 1.8 ± 0.13; Fig. 1diii) or IGFBP-5 (1.48 ± 0.16 vs. 1.5 ± 0.46; Figs. 1div) expression at 72 h between EPA and DM treated cells. In line with its ability to suppress differentiation, palmitate significantly reduced all gene transcripts associated with differentiation when compared to DM control at 72 h; IGF-II (0.51 ± 0.07; P < 0.05), MyoD (0.16 ± 0.06; P < 0.05), myogenin (0.13 ± 0.07; P < 0.05) and IGFBP-5 (0.05 ± 0.005; P < 0.05) shown in Fig. 1di–iv respectively; indicated by +.

Having observed that EPA was able to partially rescue the inhibitory effects of palmitate on biochemical differentiation, we next assessed the impact of co-incubations of these lipids on the molecular regulators of differentiation. Co-treatment of palmitate with EPA versus palmitate alone did elevate IGF-II 1.5 fold (0.75 ± 0.15 vs. 0.51 ± 0.07; P < 0.05; Fig. 1di; indicated by #), MyoD 2.2 fold (0.35 ± 0.05 vs. 0.16 ± 0.06; P < 0.05; Fig. 1dii; indicated by #), Myogenin 3.5 fold (0.45-fold ± 0.02 vs. 0.13-fold ± 0.07; P < 0.05; Fig. 1diii; indicated by #) and IGFBP-5 9.2 fold (0.46-fold ± 0.07 vs. 0.05-fold ± 0.005; P < 0.05; Fig. 1div; indicated by #). It is important to note, however, that despite these increases in gene expression with EPA in the presence of palmitate, levels were not completely restored back to control DM levels or those of EPA alone. However, this consolidated the morphological and biochemical data, where differentiation was improved with EPA in the presence of lipotoxic palmitate but not returned to levels in DM control.

Finally, in order to determine whether Id3, an inhibitor of differentiation, may be involved in palmitate-induced suppression of differentiation, we examined its expression. As is evident in Fig. 1dv, palmitate significantly increased Id3 expression levels (5.51 ± 1.04) compared with DM (1.01 ± 0.001; P < 0.05; indicated by *) or EPA alone (0.64 ± 0.06; P < 0.05). Interestingly, when EPA was co-incubated with palmitate, there was no change in expression levels compared with palmitate alone, suggesting that rescue of differentiation by EPA does not involve suppression of Id3.

### Caspases increase with palmitate and are reduced with EPA

**Fig. 2:**
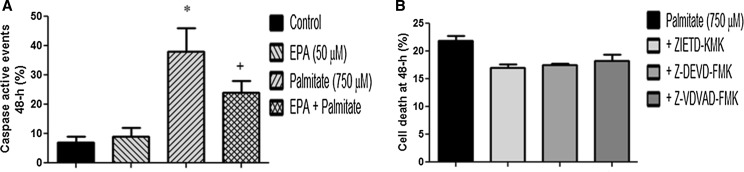
Caspases increase with palmitate and are reduced with EPA, but do not seem to be sole regulators of palmitate-induced death. **a** In control and EPA conditions, caspase activity was at baseline (7 ± 2 and 9 ± 3% respectively). The addition of palmitate to the cells significantly (P < 0.05) increased the percentage of caspase active events (38 ± 8%) (**a** indicated by *). Co-incubation of EPA and palmitate lowered caspase activity to 24 ± 4% (P < 0.05, **a** indicated by +). **b** Addition of caspase inhibitors; Z-VDVAD-FMK (caspase-2), Z-DEVD-FMK (caspasase- 3) and ZIETD-KMK (caspase 8) (**b**) only partially reduced the incidence of myoblast death in the presence of palmitate. Data are presented as Mean ± SEM (n = 3, in duplicate for each group)

To determine whether caspase activation was involved in palmitate-induced apoptosis, myoblasts were treated with a cell-permeable, FITC-conjugated, pan-caspase inhibitor (ApoStat) and increased fluorescence associated with increased caspase activity was quantified by FLOW cytometry (Fig. 2). Data illustrated that under control and EPA alone conditions, caspase activity was at baseline (7 ± 2% and 9 ± 3% respectively), consistent with the pre-G1 FLOW cytometric data described above. However, the addition of palmitate to the cells significantly (P < 0.05) increased the percentage of caspase active events (38 ± 8%) (Fig. 2a, indicated by *) initially suggesting palmitate may induce apoptosis via caspase-mediated mechanisms. Co-incubation of EPA and palmitate lowered caspase activity to 24 ± 4% (P < 0.05, Fig. 2a indicated by +). This reduction in caspase activation with EPA addition however, was in contrast to the non-significant reduction of palmitate-induced DNA fragmentation observed above (Fig. 1b).

Since caspase activity was elevated in the presence of palmitate, we wished to discover whether inhibiting caspases would reduce rates of myoblast death. We have previously demonstrated in C2 myoblasts, using western blotting and caspase activity assays that caspase-8 is activated under myotoxic (cytotoxic) stress (Stewart et al. 2004). Addition of ZIETD-FMK (caspase 8 inhibitor) in addition to Z-VDVAD-FMK (caspase-2 inhibitor) and Z-DEVD-FMK (caspase-3 inhibitor), (Fig. 2b) only partially reduced the incidence of myoblast death in the presence of palmitate, suggesting a cumulative/alternative compensatory role for these enzymes in palmitate-induced inhibition of differentiation and induction of cell death.

### MAPK inhibition reduces palmitate induced cell death

**Fig. 3:**
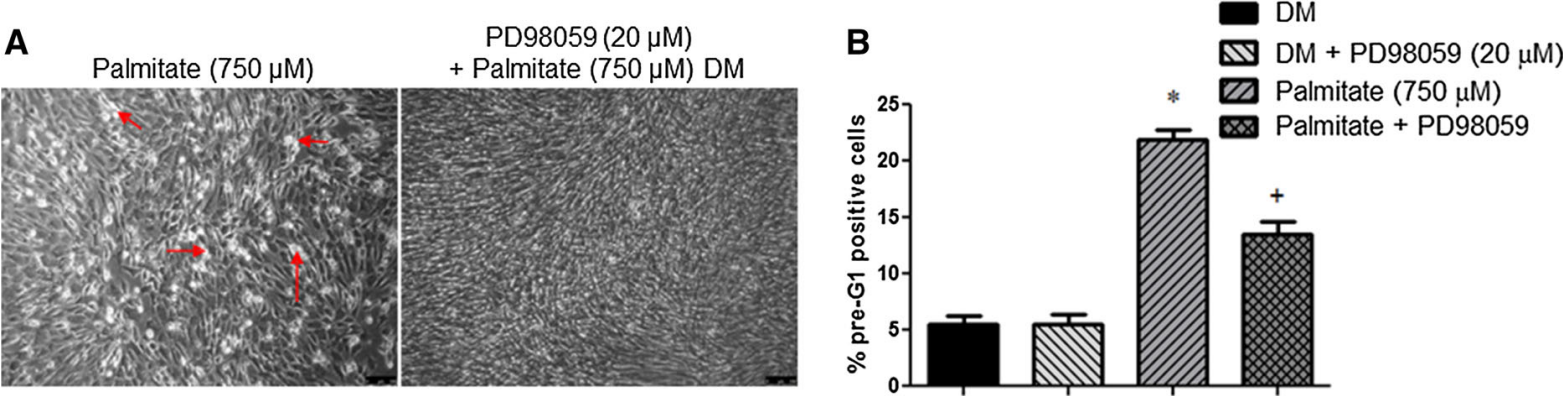
MAPK inhibition reduces Palmitate induced cell death. **a** Morphological data show that palmitate-induced death was reduced in the presence of MAPK inhibition. *Arrows* indicate congregations of dead cells. **b** Flow cytometric quantification of fragmented DNA also showed that palmitate induced apoptosis (22 ± 1%; **b** indicated by *) that was significantly reduced by co-incubation with 20 µM PD98059 (MEK inhibitor) (13.5 ± 1.2%; P < 0.05; **b** indicated by +). Data are presented as Mean ± SEM (n = 3, in duplicate for each group)

Having demonstrated that caspases are activated in the presence of palmitate, but that individual caspase inhibition does not independently rescue the effects of palmitate, we wished to investigate what other signalling pathways may be involved in the cell death effects of palmitate. We initially monitored the potential role of the MAPKs (Fig. 3a, b) in palmitate-induced apoptosis. Morphological data showed clearly that palmitate-induced cell death was reduced in the presence of MAPK inhibition (Fig. 3a). Wishing to substantiate these findings, we performed FLOW cytometric quantification of fragmented DNA and report here that the increase in apoptosis induced by palmitate (22 ± 1%; Fig. 3b, indicated by *) was significantly reduced by a co-incubation with 20 µM PD98059 (MEK inhibitor) (13.5 ± 1.2%; P < 0.05; Fig. 3b, indicated by +), confirming the morphological data in Fig. 3a. These data suggest that MAPK activation may contribute to palmitate-induced apoptosis.

### Effects of palmitate plus EPA in the presence of TNF-α and IGF-I on skeletal myoblast death and differentiation

**Fig. 4:**
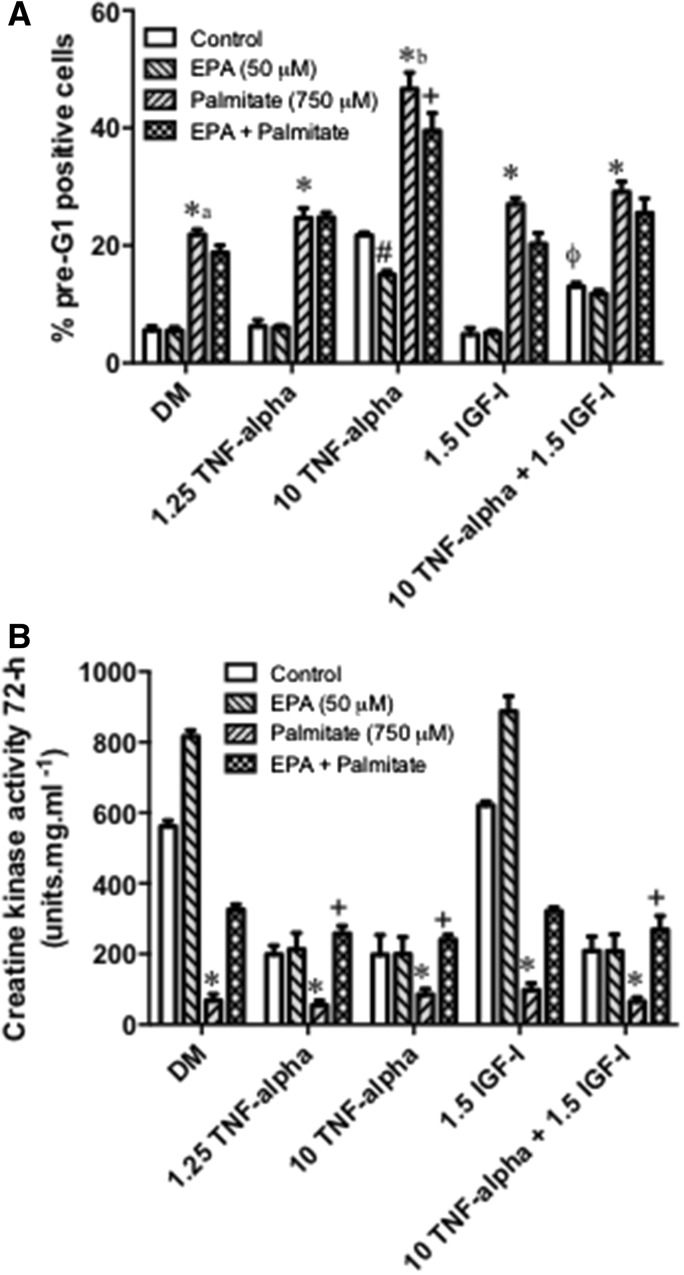
Effects of an interaction between Palmitate and EPA in the presence of TNF-α and IGF-I on skeletal myoblast death and differentiation. **a** Apoptosis: TNF-α induced apoptotic cell death in a dose dependent manner and the capacity of cells to differentiate and form myotubes was also blocked (P < 0.05) at all doses of TNF-α, as suggested previously (Foulstone et al. 2004; Saini et al. 2008; Sharples et al. 2010). With IGF-I addition (1.5 ng ml^−1^) we were able to reduce the apoptotic effect of high dose TNF-α (10 ng ml^−1^) from 22 ± 0.5 to 12 ± 0.8%; P < 0.05, **a** indicated by φ. Addition of EPA (50 µM) to high dose TNF-α (10 ng ml^−1^) significantly lowered the incidence of death (22 ± 0.5 to 15 ± 0.7% TNF-α vs. TNF-α/EPA; P < 0.05; **a** indicated by #). In contrast, palmitate addition increased myoblast death under all conditions (**a** indicated by *). As already demonstrated and confirmed here, palmitate elicited a significant increase in cell death compared to DM (5.6 ± 0.7 to 22 ± 0.9% DM vs. palmitate, P < 0.05, indicated by * **a**). Addition of palmitate to high dose TNF-α alone (10 ng ml^−1^) acted to further the incidence of death, elevating from 22 ± 0.5% in TNF-α alone to 46.6 ± 2.8% TNF-α + palmitate (P < 0.05; **a** indicated by * **b**). Addition of EPA lowered the induction of death in the presence of palmitate plus TNF-α alone from 46.6 ± 2.8 to 39.5 ± 3% (P < 0.05; a indicated by +). **b** CK activity/differentiation: As previously demonstrated (Fig. 1c), EPA was able to somewhat rescue the negative impact of palmitate on CK activity. However, EPA administration was unable to rescue the block of differentiation in myoblasts administered with TNF-α (1.25 or 10 ng ml^−1^). Palmitate (750 µM) significantly (P < 0.05) lowered the rates of CK activity under all treatment regimens ( indicated by *). Administration with EPA in the presence of palmitate could not restore the block of differentiation, but critically, in the presence of TNF-α (regardless of the treatment regime), EPA returned CK activity levels to those seen in the complete absence of palmitate e.g. it negated the interaction of palmitate plus TNF-α on differentiation (**b** indicated by +). Data are presented as Mean ± SEM (n = 3, in duplicate for each group)

Having established a potential beneficial role of EPA against palmitate-induced reductions in differentiation, we next wished to test the specificity of this finding. We have previously shown (Foulstone et al. 2004; Saini et al. 2008) and demonstrate again here (Fig. 4a) that TNF-α induces dose responsive apoptotic cell death in C2 skeletal myoblasts, with the greatest induction of cell death apparent following administration of 10 ng ml^−1^ TNF-α (22 ± 0.5%; P < 0.05). Critically, the capacity of the cells to differentiate and to form myotubes was also blocked (P < 0.05) at both doses of TNF-α tested, even that which did not induce apoptosis (1.25 ng ml^−1^; Fig. 4ai). With IGF-I addition (1.5 ng ml^−1^) we were able to reduce the apoptotic effect of high dose TNF-α (10 ng ml^−1^) from 22 ± 0.5 to 12 ± 0.8%; P < 0.05, Fig. 4a, indicated by φ.

Pre-treatment with EPA (50 µM) or palmitate (750 µM) induced opposing effects upon TNF-α induced myoblast death. Addition of EPA to high dose TNF-α (10 ng ml^−1^) significantly lowered the incidence of death (22 ± 0.5 to 15 ± 0.7% TNF-α vs. TNF-α + EPA; P < 0.05; Fig. 4a, indicated by #). In contrast, palmitate addition increased myoblast death under all conditions (Fig. 4a, indicated by *). As already demonstrated and confirmed here, palmitate elicited a significant increase in cell death compared to DM (5.6 ± 0.7–22 ± 0.9% DM vs. palmitate, P < 0.05, indicated by *a). Addition of palmitate to high dose TNF-α alone (10 ng ml^−1^) acted to further the incidence of cell death, elevating from 22 ± 0.5% in TNF-α alone to 46.6 ± 2.8% TNF-α + palmitate (P < 0.05, Fig. 4, indicated by *b). Since palmitate induced a significant increase in cell death and EPA protected myoblasts from the effects of high dose TNF-α (10 ng ml^−1^) we wished to determine whether EPA could rescue the detrimental impact of palmitate co-administration with high dose TNF-α (10 ng ml^−1^). Addition of EPA was able to lower the induction of death in the presence of palmitate plus TNF-α from 46.6 ± 2.8 to 39.5 ± 3% (P < 0.05, Fig. 4a, indicated by +).

Having determined that EPA was able to elicit a significant effect on the cell death inducing abilities of palmitate plus TNF-α, we next wished to investigate the impact of these co-incubations on myoblast differentiation. As previously demonstrated (Fig. 1c), EPA was able to increase basal differentiation and also partially rescue the negative impact of palmitate on CK activity (Fig. 4b). EPA administration was unable to rescue the block of differentiation in myoblasts administered with TNF-α (1.25 or 10 ng ml^−1^). Palmitate (750 µM) significantly (P < 0.05) lowered CK activity under all treatment regimens (Fig. 4, indicated by *). Administration with EPA in the presence of palmitate only partially rescued the block of differentiation, but critically, in the presence of TNF-α (regardless of the treatment regime), EPA returned CK activity levels to those seen in the complete absence of palmitate e.g. it negated the interaction of palmitate with TNF-α on differentiation (Fig. 4b, indicated by +). This is different to the outcome seen with apoptosis (Fig. 4a), where cell death levels in the presence of EPA did not reduce to trigger levels in the absence of palmitate, suggesting EPA is more potent at protecting against palmitate or palmitate/TNF-α induced block on differentiation rather than the induction of cell death.

### EPA enables a partial rescue of IGF-II, IGFBP-5, MyoD, myogenin and Id3 expression in the presence of both TNF-α and palmitate

**Fig. 5:**
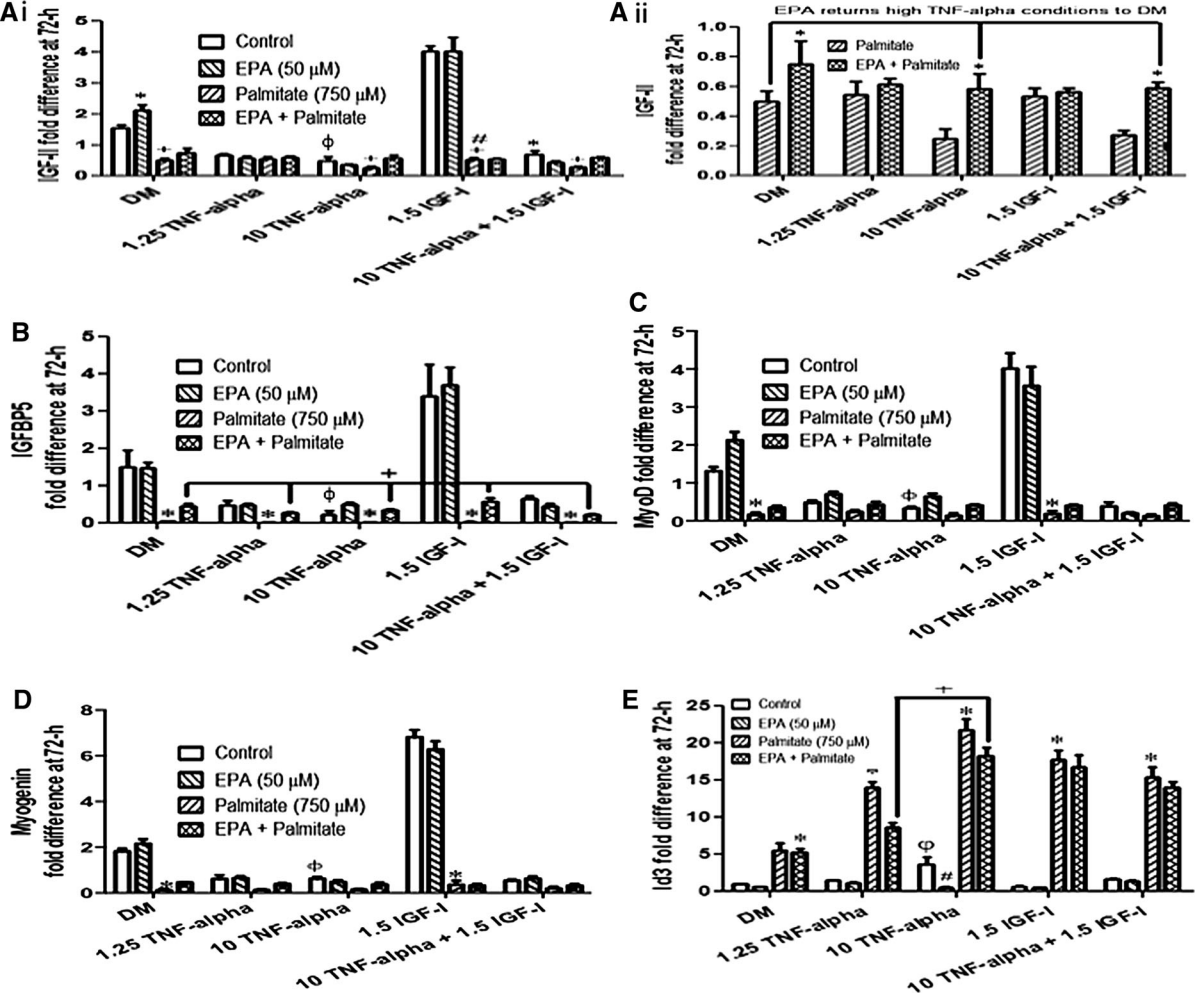
EPA enables a partial rescue of IGF-II, IGFBP-5, MyoD, myogenin and Id3 expression in the presence of both palmitate and TNF-α combined. IGF-II (**ai**, **ii**) IGFBP-5 (**b**) MyoD (**c**) and myogenin (**d**) expression was reduced, relative to control conditions in the presence of TNF-α across the dose range (highlighted by φ with high TNF-α 10 ng ml^−1^). In contrast, Id3 expression was significantly increased in myoblasts receiving high dose TNF-α relative to DM (P < 0.05, **e** indicated by φ). Addition of EPA (50 µM) induced significant (P < 0.05) increases in expression of IGF-II vs. DM alone (**ai**, indicated by *). Pre-administration with palmitate (750 µM) significantly (P < 0.05) reduced expression of IGF-II with TNF-α administration (**ai**, indicated by +). A decrease in IGF-II expression in the presence of palmitate plus IGF-I (1.5 ng ml^−1^) was also observed (P < 0.05; **ai**, indicated by #). Simultaneous co-incubation of EPA with palmitate (750 µM) was able to ameliorate some of the negative effects of palmitate alone (**aii**, indicated by *), in particular those decreases associated with high TNF-α, where levels were returned to control values in the presence of palmitate (**aii** indicated by no significant differences between high TNF-α conditions and DM i.e. P > 0.05). Addition of palmitate to myoblasts significantly reduced IGFBP-5, myoD and myogenin gene expression versus control (**b**–**d** respectively indicated by *). Co-incubation with EPA increased levels of these genes relative to palmitate alone (resulting in significant increases in IGFBP5, **b** indicated by +). These were however, still below control conditions in the presence of DM alone suggesting a partial recovery. The addition of EPA significantly reduced Id3 expression relative to high dose TNF-α (**e** indicated by #). With palmitate administration Id3 expression was greatly elevated relative to controls and peaked with high dose TNF-α alone (**e** indicated by *). Co- incubation of EPA with palmitate resulted in a decrease in Id3 expression for low (1.25 ng ml^−1^) and high dose TNF-α (10 ng ml^−1^) (P < 0.05, **e** indicated by +). Data are presented as Mean ± SEM (n = 3, in duplicate for each group)

IGF-II (Fig. 5ai, aii), IGFBP-5 (Fig. 5b), MyoD (Fig. 5c) and myogenin (Fig. 5d) expression were all down-regulated, relative to control conditions in the presence of TNF-α (indicated by φ). By contrast, Id3 expression was significantly increased in myoblasts receiving high dose TNF-α (10 ng ml^−1^) 3.6 ± 0.99 at 72-h (P < 0.05) relative to DM (Fig. 5e, indicated by φ). Addition of EPA (50 µM) induced significant (P < 0.05) increases in expression of IGF-II vs. DM alone; 2.2 ± 0.2 vs. 1.5 ± 0.1 (Fig. 5ai, indicated by *). Pre-administration with palmitate (750 µM) significantly (P < 0.05) reduced expression of IGF-II across the dose range with exception of low dose TNF-α (Fig. 5ai, indicated by +). Critically, the greatest decline in IGF-II expression in the presence of palmitate was in myoblasts administered with IGF-I (1.5 ng ml^−1^) alone where an 87 ± 7% decrease in expression was observed (P < 0.05), suggesting palmitate is capable of preventing the beneficial impact of exogenous IGF-I on endogenous IGF-II production (Fig. 5ai, indicated by #). As previously suggested, simultaneous co-incubation of EPA (50 µM) with palmitate (750 µM) was able to ameliorate some of the negative effects of palmitate alone on IGF-II expression (Fig. 5aii, indicated by *), in particular those decreases associated with high TNF-α plus palmitate, where the addition of EPA returned IGF-II expression to DM control values in the presence of palmitate alone (Fig. 5aii indicated by no significant differences i.e. P > 0.05).

Similar profiles were observed for IGFBP-5 (Fig. 5b), MyoD (Fig. 5c) and Myogenin (Fig. 5d) expression. Addition of palmitate to myoblasts significantly reduced IGFBP-5 expression across all treatments of TNF/IGF administration (to 0.034 ± 0.008 on average across all treatment groups, Fig. 5b, indicated by *). With co-incubation of EPA, IGFBP-5 levels increased significantly to 0.36 ± 0.057 (Fig. 5b, indicated by +) relative to control. For MyoD expression (Fig. 5c), palmitate addition lowered levels to 0.17 ± 0.06 on average (Fig. 5c, indicated by *) and co-incubation with EPA increased levels to 0.404 ± 0.046 relative to control conditions and resulting in increases versus palmitate alone conditions (Fig. 5c). For myogenin expression (Fig. 5d) palmitate addition lowered levels to 0.19 ± 0.04 on average (Fig. 5d, indicated by *) but co-incubation with EPA increased levels to 0.37 ± 0.025 relative to control resulting in increased expression versus palmitate alone conditions (Fig. 5d). However, these were still below control conditions in the presence of DM alone suggesting a partial recovery only above palmitate alone, yet not restoring back to baseline control levels.

The addition of EPA significantly reduced Id3 expression relative to high dose TNF-α (10 ng ml^−1^) (from 3.6 ± 0.99 to 0.55 ± 0.068 in TNF-α vs. TNF-α/EPA, respectively, Fig. 5e, indicated by *#*) suggesting EPA may stimulate differentiation by increasing expression of myogenic markers for differentiation, while simultaneously lowering expression of inhibitors of differentiation. With palmitate administration Id3 expression was greatly elevated relative to matched-controls and peaked with high dose TNF-α alone (22 ± 1.5, Fig. 5e, indicated by *). Co- incubation of EPA with palmitate resulted in a decrease in Id3 expression across the dose range and this was significant (P < 0.05) for low (1.25 ng ml^−1^) and high dose TNF-α (10 ng ml^−1^) (Fig. 5e indicated by +).

### MAPK inhibition reduces cell death in palmitate combined with elevated TNF-α conditions

**Fig. 6:**
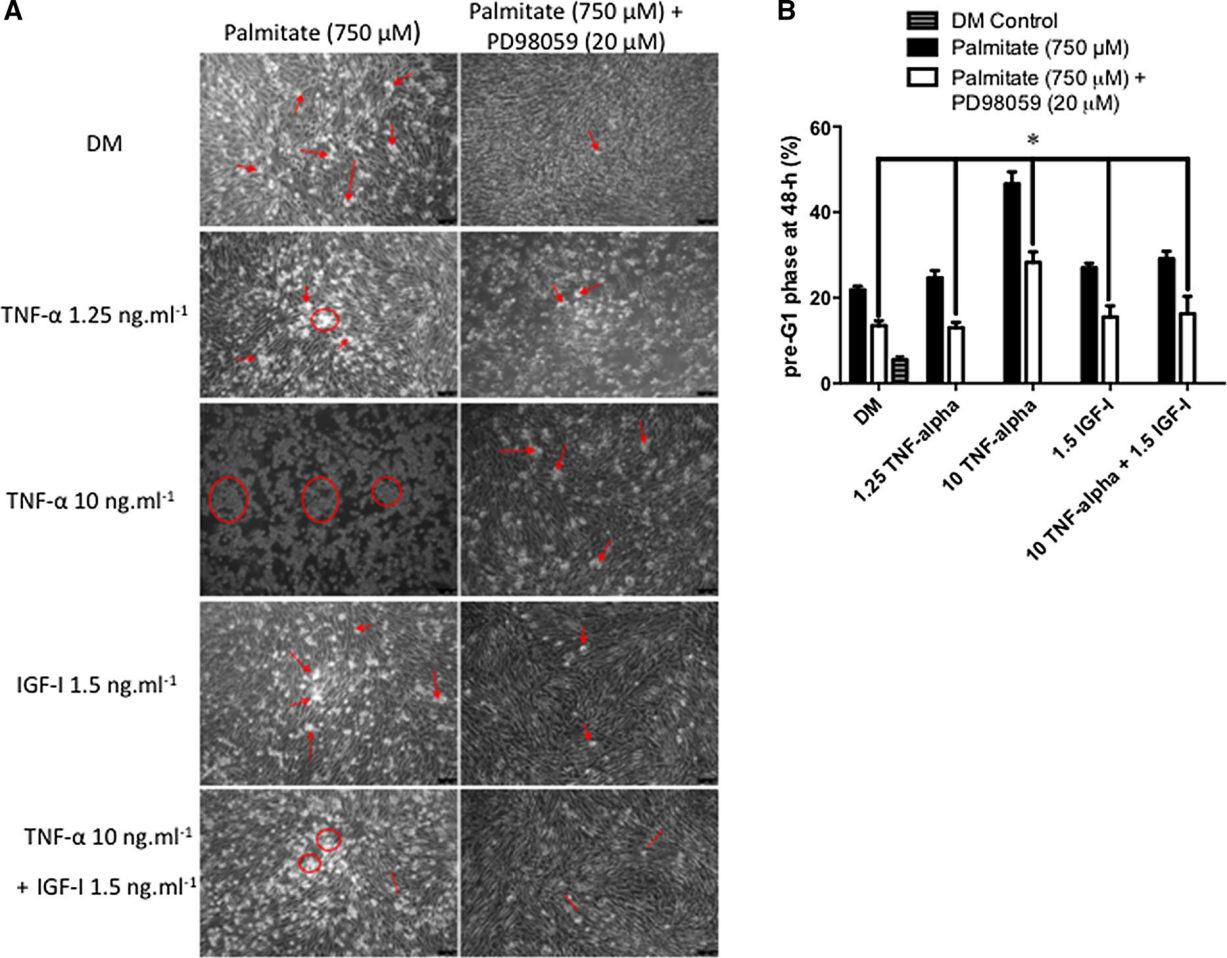
MAPK inhibition reduces cell death in palmitate combined with elevated TNF-α conditions. **a** Morphological data showed that MAPK inhibition reduced the number of detached cells in the presence of palmitate alone and in combination with TNF-α (note DM images are the same as Fig. 3a to enable a comparison with the new dosing conditions). *Arrows* indicate congregations of dead cells. **b** MAPK inhibition resulted in significant decreases in the level of apoptosis across the dose range of TNF-α (**b** indicated by *). Conditions associated with highest apoptotic death TNF-α (10 ng ml^−1^) plus palmitate were reduced from 47 ± 6 to 28 ± 2% (P < 0.05) with inhibition of MAPK’s via PD98059 20 µM. Data are presented as Mean ± SEM (n = 3, in duplicate for each group)

Having demonstrated that MAP Kinase activation was important for the induction of cell death in the presence of TNF-α or palmitate alone, we next wished to investigate whether MAP Kinases underpinned the enhanced apoptotic potential of palmitate combined with TNF-α. Morphological data showed clearly that MAPK inhibition reduced the number of detached cells (Fig. 6a) and this reduction resulted in a decrease in the level of apoptosis (Fig. 6b). As shown above, when compared with controls, apoptosis was significantly elevated across the treatment conditions in the presence of palmitate alone, low dose and high dose TNF-α. Inhibition of MEKK signaling with PD98059 (20 µM) was effective in lowering rates of cell death observed in palmitate conditions to levels comparable to control conditions, where basal cell death following MAPK inhibition averaged 13 ± 3% for DM, low dose TNF-α and IGF-I alone. Interestingly, in conditions associated with highest apoptotic death, TNF-α (10 ng ml^−1^) plus palmitate, apoptosis was reduced from 47 ± 6 to 28 ± 2% (P < 0.05) following inhibition of MAPK activity via PD98059 administration (Fig. 6a, b indicated by *).

### Ceramide inhibition reduces cell death in palmitate plus TNF-α conditions via JNK signaling

Finally, we also wished to discover the potential involvement of ceramide, one of the members of the sphingomyelin family and an important component of the lipid bilayer. Ceramide has been reported to be increased following palmitate administration (Listenberger et al. 2001), with TNF-α also regulating its production and controlling its downstream signaling (De Larichaudy et al. 2012) via the MAP kinases to induce apoptosis (Stewart et al. 1999a, b). FumonisinB1 (FB1; 20 µM), which blocks ceramide synthase and thereby ceramide accumulation, was added in the absence or presence of palmitate and/or high dose TNF-α (10 ng ml^−1^). Furthermore, since JNK signaling is linked to ceramide activity and apoptosis we also measured JNK activity using cytometric bead array technology. The objective was to discover if ceramide and/or JNK were central to the role of palmitate/TNF-α induced myoblast death using an inhibitor that was specific to the sphingomyelin pathway (N.B. at 20 µM FB1 does not inhibit known protein phosphatases).

**Fig. 7:**
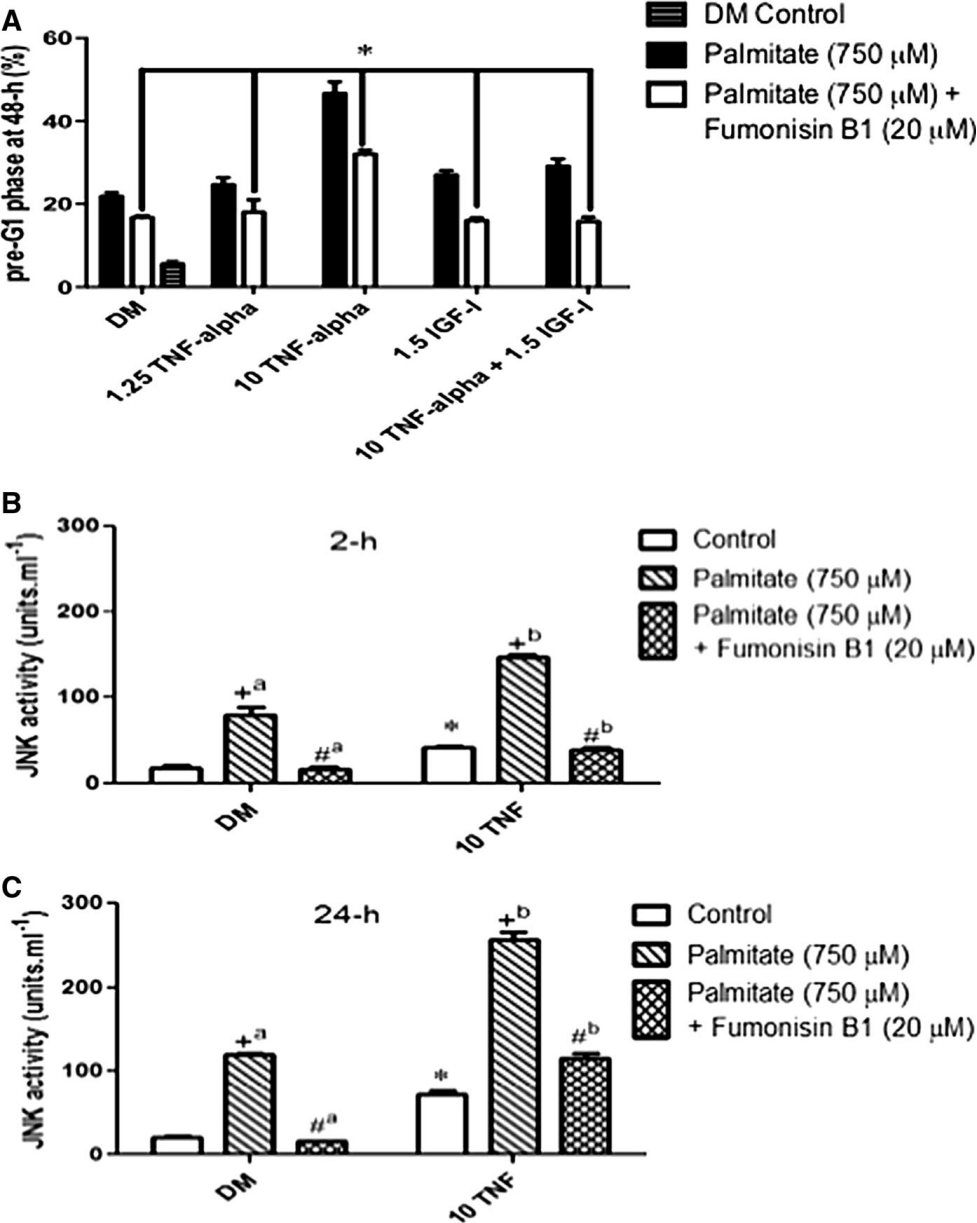
Ceramide inhibition reduces cell death with palmitate and TNF-α via JNK signaling. **a** Administration of FB1 (20 µM) was effective at reducing the incidence of myoblast death induced by palmitate (**a** indicated by *) across the dose range for TNF-α. Maximal apoptotic death with high dose palmitate plus TNF-α (10 ng ml^−1^) alone was lowered from 47 ± 6 to 32 ± 2% by a pre-incubation with FB1. **b**, **c** JNK activity in the absence or presence of palmitate plus/minus TNF-α at 2-h **b** and 24-h **c**. JNK activity was significantly elevated with high dose TNF-α administration at 2-h (P < 0.05, 41.5 ± 2.5 units ml^−1^ **b** indicated by *) and at 24-h (72 ± 4 units ml^−1^, c indicated by *) versus DM control. With palmitate alone, a significant (P < 0.05) elevation in JNK activity was observed (79.5 ± 9.5 units ml^−1^, **b** indicated by +^a^) at 2-h and 119.5 ± 2.5 units ml^−1^ at 24-h, **c** indicated by +^a^). JNK activity continued to rise to 24-h and maximal levels were reported with high dose TNF-α plus palmitate at 256 ± 10 units ml^−1^ (**c** indicated by +^b^). Following co-treatment of palmitate with FB1 a decrease in JNK activity was observed across the dose conditions with levels returning to basal in myoblasts incubated in DM alone, ~17 ± 1 units ml^−1^ (Fig. 8b, c indicated by #^a^). With conditions that induced maximal apoptotic death (high 10 TNF-α), co-treatment of palmitate with FB1 lowered rates of death and this was mirrored by significant reductions in JNK activity both at 2-h (**b** indicated by #^b^)and 24-h (**c** indicated by #^b^) back to those of basal levels. Data are presented as Mean ± SEM (n = 3, in duplicate for each group)

Pre-administration of FB1 (20 µM) appeared effective at reducing the incidence of myoblast death induced by palmitate (Fig. 7a, indicated by *), where cell death was still significantly below counterparts administered with palmitate alone in the absence of FB1 (Fig. 7a; P < 0.05 for all treatments, indicated by *). Maximal apoptotic death with high dose palmitate plus TNF-α (10 ng ml^−1^) alone was lowered from 47 ± 6% in palmitate alone conditions to 32 ± 2% when FB1 was administered in the presence of palmitate.

**Fig. 8:**
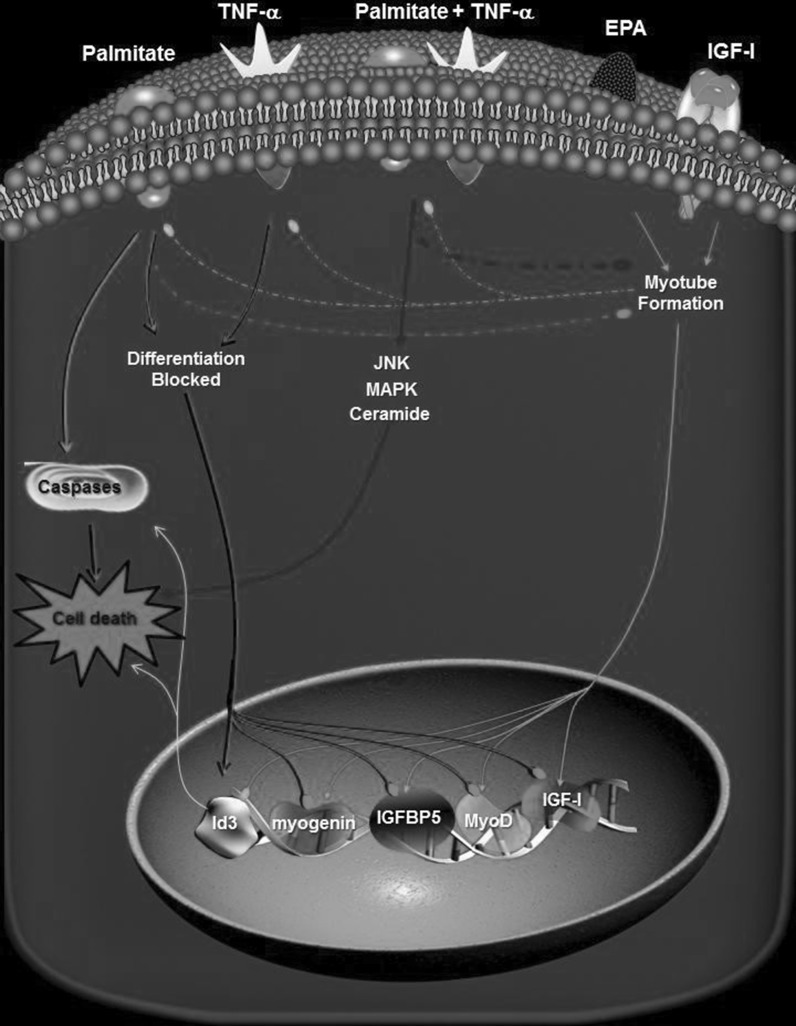
Myoblast signaling associated with EPA, Palmitate and TNF-α interaction. Palmitate induces lipotoxicity in skeletal myoblasts through activation of caspase cascades resulting in cell death and inhibition of differentiation by inducing transcriptional repression of pro-myogenic transcription factors MyoD, myogenin, IGF-II and IGFBP5 whilst activating Id3 associated with inhibition of differentiation, caspase activation and cell death. Palmitate alone or palmitate with TNF-α inhibits IGF signalling. Palmitate in combination with cytokine, TNF-α, induces both lipotoxic and cytotoxic inflammatory processes resulting in the activation of MAPK, ceramide and JNK pathways that are associated with cell death processes. Signal inhibition of respective pathways using PD98059 and Fumonisin B1 induced survival. It is proposed lipotoxicity associated with palmitate in the present study represents a myoblast muscle model of obesity resulting in impaired regenerative capacity of skeletal muscle. Combined TNF-α and palmitate represents a proposed model of sarcopenic obesity whereby chronic elevated inflammation is associated with sarcopenia and palmitate associated with increased circulating fatty acids associated with obesity. EPA administration is able to partially stimulate differentiation with palmitate alone suggesting in an lipotoxic ‘obese’ environment EPA can enable myotube formation. In a cytotoxic and lipotoxic ‘sarcopenic obese’ environment EPA can induce partial rescue of death and differentiation suggesting rescue of death may be due to dampening on inflammatory processes and myotube formation due to dampening of lipotoxic processes. EPA also downregulates Id3 expression and correlates with reduced cell death under conditions of lipotoxicity and cytotoxicity. Thus EPA has potential therapeutic potential in both obese and sarcopenic obese individuals

Finally, we specifically looked at the effect that this inhibitor had on JNK activity in treatments that induced maximal cell death, palmitate with high dose TNF-α 10 ng ml^−1^ (Fig. 7b, c). In control conditions basal JNK activity with DM control treatment at 2- and 24-h was approximately 20 ± 1 units ml^−1^ (Fig. 7b, c, indicated by *). This was elevated with high dose TNF-α administration with levels being significantly greater (P < 0.05) at 24-h (72 ± 4 units ml^−1^, Fig. 7c, indicated by *) compared with 2-h (41.5 ± 2.5 units ml^−1^, Fig. 7b, indicated by *). Following pre-treatment with palmitate, a significant (P < 0.05) elevation in JNK activity was observed, even under basal conditions (79.5 ± 9.5 units ml^−1^, Fig. 7b, indicated by +^a^ at 2-h and 119.5 ± 2.5 units ml^−1^ at 24-h, Fig. 7c, indicated by +^a^), suggesting palmitate alone induces JNK activation. JNK activity continued to rise to 24-h and maximal levels were reported with high dose TNF-α plus palmitate at 256 ± 10 units ml^−1^ (Fig. 7c, indicated by +^b^). Following co-treatment of palmitate with FB1 a decrease in JNK activity was observed across the conditions with levels returning to basal in myoblasts incubated in DM alone, ~17 ± 1 units ml^−1^ (Fig. 8b, c, indicated by #^a^). With conditions that induced maximal apoptotic death high TNF-α and co-treatment of palmitate, incubation with FB1 lowered rates of cell death and this was mirrored by significant reductions in JNK activity both at 2-h (Fig. 7b, indicated by #^b^) and 24-h (Fig. 7c, indicated by #^b^) back to those of basal levels. Most notably at 24-h FB1 decreased JNK activity by 66 ± 7% in high dose TNF-α conditions in the presence of palmitate.

## Discussion

In older individuals, age-related changes in body composition, as well as the increased prevalence of obesity, determine a combination of excess weight and reduced muscle mass or strength, recently defined as sarcopenic obesity (SO). Circulating FFAs and pro- inflammatory cytokines are elevated and potentially represent a causal link between obesity and muscle wasting. Palmitate (saturated FFA) is associated with reduced IGF signalling—a key pathway in skeletal muscle differentiation whereas eicosapentaenoic acid (EPA) a-3 polyunsaturated fatty acid with demonstrable anti-inflammatory activities may have potential benefits with regards to atrophic skeletal muscle conditions. Terminally differentiated myofibres are incapable of division (Saini et al. 2009). Following damage mononucleated stem cells proliferate, differentiate and enable repair (Saini et al. 2009). We have investigated the effects of palmitate and EPA on differentiating muscle using undifferentiated murine C2 myoblasts. Additionally, the effect of these FFAs on cell death or regeneration was investigated in cell models, previously shown to block differentiation using low and high level cytokine stress e.g. TNF (1.25 and 10 ng ml^−1^) and to induce cell death (10 ng ml^−1^ TNF). The effects of these particular FFAs on muscle cells have previously been shown to have either adverse or therapeutic effects, respectively (Magee et al. 2008; Sabin et al. 2007). In vitro studies have demonstrated that palmitate reduces insulin-stimulated phosphorylation of AKT (pAKT) (Sabin et al. 2007)—a key downstream signalling molecule in the IGF system which acts potently to regulate protein synthesis, skeletal muscle regeneration and differentiation (Glass 2003) whereas EPA is able to reduce TNF-α-mediated loss of myosin heavy chain expression and increase myogenic fusion (Magee et al. 2008), although the downstream mechanisms remained to be defined.

### Impact of FFAs, TNF-α and IGF-I on skeletal muscle cell death and differentiation

In the present study, palmitate (750 µM) was associated with an increase in apoptosis and inhibition of differentiation in all conditions tested. Addition of palmitate to cyotoxic conditions, high dose TNF-α alone (10 ng ml^−1^) acted to further the incidence of cell death. Biochemical and gene expression analyses for markers of differentiation showed a decline in skeletal myoblast creatine kinase activity and IGF-II, IGFBP5, MyoD and myogenin expression with palmitate pre-treatment. Furthermore, Id3 which functions as a negative regulator of differentiation by integrating mitogenic growth factor signalling into the gene regulatory programme maintaining cell cycle progression, was highly expressed when myoblasts were administered with palmitate. Critically, elevated Id3 expression is also associated with increased death in myoblasts (Atherton et al. 1996). By contrast, EPA (50 µM) treatment reduced incidence of myoblast death and in conditions that induced myotube formation EPA enhanced this response. Addition of EPA to high dose TNF-α (10 ng ml^−1^) significantly lowered the incidence of cell death compared with either treatment in the absence of EPA and simultaneously increased biochemical and morphological differentiation, which were reflected by significant increases in expression of IGF-II, IGFBP-5 and Myogenin.

Having demonstrated opposing effects of EPA (50 µM) and palmitate (750 µM) upon myoblast death, specifically EPA protecting myoblasts from the effects of high dose TNF-α (10 ng ml^−1^) or palmitate-induced reductions in differentiation, we wished to discover if co-incubating EPA with Palmitate could potentially ameliorate the cyotoxic effects and stimulate myotube formation in high dose TNF-α (10 ng ml^−1^) as well as low dose TNF-α (1.25 ng ml^−1^) co-incubated with IGF-I compared with palmitate alone. Addition of EPA was able to lower the induction of cell death in the presence of palmitate plus TNF-α. Despite this protective effect, the incidence of cell death was still significantly higher than in the presence of any of the myotoxic triggers alone, suggesting the activation of pathways resistant to the protective effects of EPA treatment. Having determined that EPA was able to elicit a small but significant effect on the cell death inducing abilities of palmitate plus TNF-α, we next wished to investigate the impact of these co-incubations on myoblast fusion. Co-incuation of palmitate and EPA, elevated creatine kinase activity compared with palmitate alone suggesting EPA stimulates differentiation. Critically, in the presence of TNF-α (regardless of the treatment regime), EPA returned CK activity levels to those seen in the complete absence of palmitate e.g. it negated the interaction of palmitate with TNF-α on differentiation. This is different to the outcome seen with apoptosis, where cell death levels in the presence of EPA did not reduce to trigger levels in the absence of palmitate, suggesting EPA is more potent at protecting against the palmitate/TNF-α-induced block on differentiation than induction of cell death.

In parallel with increased creatine kinase activity co-treatment of palmitate with EPA also elevated IGF-II, IGFBP-5, MyoD, Myogenin but did not suppress the expression of Id3. The findings suggest EPA is capable of partially rescuing the inhibitory impact of palmitate on differentiation potentially via genes associated with myotube formation but does not involve suppression of Id3 at the time-points investigated. Although gene expression, biochemical and morphological analyses were conducted up to 72 h, it is possible that measurement of markers of differentiation at 96- and 120-h may have been rescued at these latter time points with addition of EPA to palmitate. Nonetheless, this would have indicated that the capacity to form myotubes is delayed/impeded in the context of regenerating muscle.

### Mechanisms for palmitate-induced cell death associated with TNF-α and IGF-I interaction in skeletal myoblasts

Cytotoxic accumulation of long chain fatty acids have been proposed to play an important role in the pathogenesis of diabetes mellitus, heart disease and potentially sarcopenic obestiy. To explore the mechanism of cellular lipotoxicity, studies by Listenberger et al. (Listenberger et al. 2001) have demonstrated in the presence of media supplemented with fatty acid, the saturated fatty acid palmitate, induces programmed cell death as determined by annexin V positivity, caspase 3 activity, and DNA laddering. We determined whether caspase activation was involved in the cell death effect of palmitate addition. Whilst the addition of palmitate significantly increased (P < 0.05) caspase activity, indicating that palmitate may induce cell death via a caspase mediated mechanism, EPA only partially reduced the impact of palmitate on caspase activation and caspase inhibition where the use of Z- VDVAD-FMK (caspase), Z-DEVD-FMK (caspase), Z-IETD-FMK (caspase 8) inhibitors only partially reduced the incidence of myoblast death. Interestingly, it is reported that Id-3 is capable of inducing apoptosis via caspase-mediated pathways (Kee 2005; Norton and Atherton 1998; Simbulan-Rosenthal et al. 2006). The inability of EPA to significantly reduce palmitate-induced Id-3 expression, may be linked to sustained caspase activation and maintained apoptosis in the presence of palmitate plus EPA.

Having demonstrated that EPA does not reduce Id-3 expression or DNA fragmentation and that individual caspase inhibition did not significantly reduce myoblast death following palmitate pre-treament, we explored whether alternative signalling pathways may be involved. Previously we have suggested a novel role for MAP kinase in ceramide-induced apoptosis as well as the up-regulation of the c-Jun N terminal-kinase (JNK) signalling pathway in TNF-α-induced cell death (Stewart et al. 1999b), palmitate uptake (Sabin et al. 2007) and myoblast models of ageing (Sharples et al. 2011). We therefore wished to investigate whether MAPKs and JNK were involved in palmitate mediated apoptosis in the present model.

Specifically, we have shown that C2, C2C12 and human skeletal myoblasts are susceptible to apoptotic death, in a dose responsive fashion to TNF-α (Foulstone et al. 2001, 2004; Saini et al. 2008; Sharples et al. 2010; Stewart et al. 2004). In C2 cells, optimal cyotoxicity was identified at 10 ng ml^−1^ TNF-α. The capacity of the cells to differentiate and to form myotubes was blocked even at 1.25 ng ml^−1^ TNF-α which does not induce apoptosis (Saini et al. 2008). Co-incubation with IGF-I (1.5 ng ml^−1^) was able to reduce the apoptotic effects of high dose TNF-α (10 ng ml^−1^) but not rescue the block on differentiation. Myotube formation and creatine kinase activity were also blocked by 1.25 ng ml^−1^ TNF-α (Saini et al. 2008). As predicted, media supplemented with palmitate significantly elevated cell death across the treatment groups: DM, low dose TNF-α and IGF-I alone. This was further elevated in conditions where apoptosis was already elevated (high dose TNF-α) causing an increase in cell death. This increase was however, blocked by a pre-incubation with PD98059 (20 µM). Although MAPK activity was not assessed directly, we have previously reported a reduction in MEK-ERK1/2 activation following PD98059 administration at these doses and in this model (Al-Shanti and Stewart 2008; Alessi et al. 1995; Saini et al. 2008). Where PD98059 in palmitate samples was effective in lowering rates of cell death to levels comparable to control conditions. Basal cell death following MAPK inhibition averaged 13 ± 3% for DM, low dose TNF and IGF-I alone. In conditions associated with maximal apoptotic death (high dose TNF) was reduced to 28 ± 2%. The above findings therefore suggest MAPK to be a central component for the induction of apoptosis under cytotoxic and/or lipotoxic conditions.

Evidence also suggests the role of ceramide functioning downstream of palmitate and TNF-α but upstream of the MAPK pathway, which potentially acts as a master regulator of lipid- and cytokine-induced apoptosis. Intracellular accumulation of long chain fatty acids in non- adipose tissues is associated with cellular dysfunction and cell death and may ultimately contribute to the pathogenesis of disease (Listenberger et al. 2001). It has been hypothesised that FFAs are precursors for *de novo* ceramide synthesis, it has also been hypothesised that fatty acid- induced apoptosis occurs through this pathway. Indeed, studies have previously shown that saturated fatty acids induce *de novo* ceramide accumulation and apoptosis in cultured myotubes (Turpin et al. 2006). Extensive evidence shows that saturated fatty acids can induce apoptosis in several cell types including pancreatic β-cells, hepatocytes, cardiomyocytes and skeletal muscle myotubes in vitro *(*Hickson-Bick et al. 2000; Shimabukuro et al. 1998; Turpin et al. 2006; Wei et al. 2006). Fatty acid-induced apoptosis, or ‘lipo-apoptosis’, causes changes in tissue function. Ceramide is a lipid second messenger involved in the apoptotic response induced by TNF-α, ionising radiation and heat shock (Kolesnick and Kronke 1998). These stimuli are thought to increase ceramide by hydrolysis of sphingomyelin rather than *de novo* biosynthesis. The downstream signalling pathways through which ceramide initiates apoptosis remain unclear, but several possible components have been identified. Direct targets of ceramide include ceramide-activated protein kinase (CAPK, KSR), protein kinase C, and ceramide-activated protein phosphatase (Mathias et al. 1998). Ceramide signalling is affected by mitogen- activated protein kinase (our findings support a role of the MAPK system in myoblast apoptosis) and c-Jun N-terminal kinase (JNK) signalling cascades ultimately leading to growth arrest and apoptosis (Mathias et al. 1998; Obeid and Hannun 1995).

Under control conditions basal JNK activity was observed with DM treatment. In conditions where myoblast cell death was observed, high dose TNF-α administration, JNK phosphorylation was significantly elevated. Pre-treatment with palmitate further elevated JNK activity even under basal conditions, suggesting palmitate induces death through JNK phosphorylation. Following co-treatment of palmitate with FB1 (FumonisinB1 which blocks ceramide synthase and thereby ceramide accumulation) there was a reduction in the incidence of myoblast death that was accompanied by a decrease in JNK phosphorylation. Although cell death was still elevated above basal conditions for DM, low dose TNF-α alone and IGF-I alone (16 ± 1, 18 ± 3, and 16 ± 1%, respectively) this was significantly below the same treatments administered with palmitate in the absence of FB1. Maximal apoptotic death with high dose TNF-α alone (10 ng ml^−1^) had been lowered to 15 ± 4% and was accompanied by 66 ± 7% decrease in JNK activity. These findings suggest that the mechanisms by which palmitate induced death in these models is the result of ceramide and JNK activity.

Lipotoxicity is a commonly used term that broadly describes detrimental changes to cell morphology and function induced by an excess of fatty acids and/or intracellular accumulation of lipids that may cause cell death. There is no doubt that lipotoxicity, as originally defined, is evident in the pancreas, liver and possibly the heart (Garris 2005; Shimabukuro et al. 1998; Sparagna et al. 2000; Summers 2006; Wei et al. 2006). Likewise, lipid overload undoubtedly disrupts some aspects of skeletal muscle function, such as insulin signal transduction. This study was undertaken to specifically determine whether an excess of lipids causes apoptosis in skeletal muscle, and whether these events are related to changes in muscle morphology. Our studies indicate that acute fatty acid overload can promote pro- apoptotic signaling and significantly reduce the ability of myoblasts to differentiate. This therefore warrants exploration in the context of in vivo adult muscle regeneration. Fatty acid-induced apoptosis may therefore contribute to skeletal myoblast death associated with sarcopenic obesity. Palmitate induces myoblast apoptosis potentially through altered Id-3 mRNA expression, caspase activation and/or, ceramide, MAPK and JNK pathways and the inhibition of differentiation is characterized via suppression of key regulatory myogenic factors, such as myogenin, myoD, IGF-II and IGFBP-5 (Fig. 8). By contrast EPA does appear to have a protective action against MAPK mediated cell death and reduced differentiation but not against Id3/caspase-induced apoptosis (Fig. 8).

In the present study we propose that the actions of palmitate alone represent a model of the effects of obesity in regenerating skeletal muscle where increased fatty acid levels are associated with cell death and reduced differentiation, thus impacting on the regenerative capacity of muscle. Combined with the cytokine, TNF-α, we propose that the cell models represent a sarcopenic obese environment, given that chronic elevated inflammation is a characteristic of ageing muscle and increased fatty acid infiltration is a characteristic of obese muscle. As demonstrated in our study, the sarcopenic obese model induced the greatest impact on cell death and differentiation (Fig. 8).

These findings support further investigations of EPA as a potential therapeutic agent in conditions associated with elevated saturated fat, cytokine inflammation and reduced muscle mass indicative of sarcopenic obesity.
